# Bone marrow edema as an independent imaging predictor of dedifferentiated chondrosarcoma

**DOI:** 10.1186/s13018-026-07093-8

**Published:** 2026-07-15

**Authors:** Caleb Cummings, Joseph Redding, Chloe Chose, Sarah C. Tepper, David M. Joyce, Odion T. Binitie, Jamie T. Caracciolo, Alexander L. Lazarides

**Affiliations:** 1 University of South Florida Health Morsani College of Medicine, Tampa, FL 33602 USA; 2 Department of Sarcoma, H. Lee Moffitt Cancer Center, Tampa, FL 33612 USA; 3 Department of Diagnostic Imaging and Interventional Radiology, H. Lee Moffitt Cancer Center, Tampa, FL 33612 USA

**Keywords:** Dedifferentiated chondrosarcoma, Marrow edema, Magnetic resonance imaging

## Abstract

**Background:**

Dedifferentiated chondrosarcoma is the most aggressive subtype of chondrosarcoma; however, imaging features that independently distinguish it from other subtypes remain unclear. The purpose of this study was to identify independent imaging predictors of long bone dedifferentiated chondrosarcoma to signal for expedited biopsy and treatment.

**Methods:**

This retrospective study incorporated consecutive patients undergoing resection at a tertiary center for primary intramedullary long bone chondrosarcoma between February 2001 and January 2024. Inclusion required preoperative MRI and postoperative pathological confirmation. Seven imaging features were analyzed. Multivariate regression investigated independent predictors of dedifferentiated versus non-dedifferentiated chondrosarcoma.

**Results:**

Sixty-seven patients were included. Of these, 23 (34%) patients presented with dedifferentiated chondrosarcoma, while 43 (64%) had conventional chondrosarcoma (21 grade 1, 18 grade 2, 4 grade 3) and one (1.5%) had grade 3 mesenchymal chondrosarcoma. On multivariate analysis, bone marrow edema was the only independent predictor of dedifferentiated status (*p* = 0.0091). This association persisted after controlling for age, sex, and tumor size (*p* = 0.0057). Bone marrow edema achieved a sensitivity and specificity of 87% and 68%, respectively, and the greatest area under the ROC curve among individual imaging features (AUC = 0.78, *p* = 0.0027), while the overall combined model achieved even greater discrimination (AUC = 0.87, *p* < 0.001).

**Conclusions:**

Marrow edema was the only imaging feature that independently predicted dedifferentiated chondrosarcoma in multivariable analysis. Although incorporation of additional imaging findings provides the highest overall diagnostic accuracy, marrow edema alone demonstrates high sensitivity and is a readily identifiable marker that may prompt expedited biopsy when identified on routine preoperative MRI.

## Background

 Dedifferentiated chondrosarcoma is a rare but highly aggressive malignancy, associated with the poorest prognosis of all chondrosarcoma subtypes. The median survival for patients with dedifferentiated chondrosarcoma is only 11 months, significantly worse than that of grade 2 and grade 3 chondrosarcomas, which have median survival times of 100 and 32 months, respectively [1, 2]. A key contributor to this poor prognosis is the high rate of metastasis, with 21% of patients presenting with metastatic disease at the time of diagnosis [3]. Survival differs markedly based on metastatic status: patients with metastasis have a median survival of just 5 months compared to 13 months for those without [3].

Although some studies have shown slight survival improvements with chemotherapy in patients with dedifferentiated chondrosarcoma [4–6], both conventional and dedifferentiated chondrosarcomas are generally resistant to chemotherapy and radiation, making surgical resection the primary treatment modality [7–9]. However, dedifferentiated tumors demonstrate substantially more aggressive biologic behavior, more rapid progression, and significantly poorer survival than conventional grade 2 or grade 3 chondrosarcoma. Given these poor-prognostic factors, early diagnosis and prompt surgical intervention are critical to improving patient outcomes.

Accurate tumor characterization is essential for guiding treatment decisions. While preoperative biopsy remains the gold standard for diagnosis, recent advances in imaging have provided promising non-invasive methods of characterization. The Radiological Aggressiveness Score, for example, evaluates nine imaging features to distinguish between resection-grade chondrosarcomas (grades 2, 3, and dedifferentiated) and lower-grade or atypical cartilage tumors [10]. Another approach, the Birmingham Atypical Cartilage Tumor Imaging Protocol, aids in triaging patients for specialist referral versus nonsurgical surveillance, though it does not assign tumor grade [11]. Although several radiographic findings have been associated with dedifferentiated chondrosarcoma [12, 13], the independent predictive value of individual imaging features remains incompletely understood. Furthermore, while the Radiological Aggressiveness Score incorporates nine imaging characteristics associated with aggressive cartilage tumors, it was developed to distinguish resection-grade lesions broadly rather than specifically identify dedifferentiated histology. Therefore, we evaluated the individual imaging characteristics incorporated within the Radiological Aggressiveness Score to identify independent predictors of dedifferentiated chondrosarcoma.

The purpose of this study was to evaluate whether specific radiologic features can predict dedifferentiated chondrosarcoma, with the goal of providing pre-biopsy risk-stratification markers to prompt expedited referral, targeted biopsy sampling, and timely surgical planning in lesions at high risk of dedifferentiation.

## Methods

This retrospective cohort study received institutional review board approval. Consecutive patients who underwent surgical resection for a primary intramedullary chondrosarcoma of the long bones at a tertiary cancer center between February 2001 and January 2024 were included. Inclusion also required preoperative magnetic resonance imaging (MRI). The study focused exclusively on long bone lesions (excluding pelvic and axial lesions), as prior research has demonstrated distinct imaging characteristics within this subset [10]. Additionally, patients with extraosseous or periosteal chondrosarcoma were excluded due to the inconsistent behavior of these subtypes with respect to the radiologic features analyzed compared to intramedullary lesions. The outcome measure of interest was dedifferentiated or non-dedifferentiated status.

Preoperative imaging for each patient was evaluated for the presence or absence of the nine binary radiologic features used previously by the Radiological Aggressiveness Score to characterize chondrosarcoma [10]. These features included: pathologic fracture, endosteal scalloping involving more than two-thirds of the transverse circumference, endosteal scalloping involving more than two-thirds of the cranio-caudal length, medullary expansion, complete cortical breach, periosteal reaction, cortical thickening, bone marrow edema, and soft tissue involvement. Imaging features were evaluated by two trained reviewers, with any discrepancies resolved through consultation with an experienced orthopedic oncology surgeon and musculoskeletal radiologist. Marrow edema was assessed qualitatively, and no minimum thickness threshold was required. Final diagnoses were confirmed using surgical pathology following resection.

Complete cortical breach was excluded from the final analysis due to its 100% sensitivity, which caused quasi-complete separation and destabilized the multivariate model. Additionally, two features (endosteal scalloping of 2/3 circumference and endosteal scalloping of 2/3 cranio-caudal length) were merged into one common variable (> 2/3 endosteal scalloping) due to excessive collinearity. This new, combined variable required both original features to be present. These alterations resulted in a total of seven imaging features included in the final multivariate analysis (Table 1).

**Table 1:** Description of each feature on X-Ray, computer tomography and magnetic resonance imaging

Imaging feature	Description
Pathologic fracture	Full cortical breakage in an orthogonal plane
> 2/3 Endosteal Scalloping	Endosteal scalloping of > 2/3 axial circumference and 2/3 cranial-caudal length of tumor
Medullary expansion	Widening of the medullary cavity beyond normal bone contour
Periosteal reaction	New bone formation on the periosteal surface
Cortical thickening	Increased cortical width relative to adjacent bone
Marrow Edema	Ill-defined, hyperintense signal on T2/STIR MRI extending beyond tumor margin on T1 MRI
Soft-tissue involvement	Extension of tumor signal beyond the cortex, into adjacent soft tissues

Statistical analyses were performed in Python using scikit-learn, pandas, numpy, and statsmodels. We assessed discrimination by computing ROC AUCs for each individual predictor and for the combined 7-factor logistic regression model, with 95% confidence intervals derived via a stratified nonparametric bootstrap using 1,000 resamples and reporting percentile-based intervals. Welch’s t-test (age at surgery and tumor size) and chi-squared test (sex) were used to analyze differences between patients with dedifferentiated versus non-dedifferentiated chondrosarcoma. Fisher’s exact test was used to calculate association of each imaging feature with dedifferentiated chondrosarcoma on univariate analysis. A multivariate logistic regression was completed to determine the independent contribution and odds ratios of each imaging factor while controlling for all others. Variance Inflation Factors (VIFs) were used to measure collinearity between imaging features. Model calibration was assessed using the Hosmer–Lemeshow goodness-of-fit test. A receiver operating characteristic (ROC) analysis was used to calculate the area under the curve (AUC) for each individual factor, as well as the overall model, to determine each factor’s predictive capacity of dedifferentiated chondrosarcoma. DeLong’s test for correlated AUC curves was performed to analyze differences between individual imaging features versus the overall, combined 7-factor multivariate model.

## Results

Sixty-seven patients were identified who met inclusion criteria for the study. Of these patients, 43 had conventional chondrosarcomas (21 grade 1, 18 grade 2, 4 grade 3), 23 had dedifferentiated chondrosarcomas, and one had grade 3 mesenchymal chondrosarcoma. The mean age of patients at surgery was 61 years (range of 18–85 years) and consisted of 33 males and 34 females. There was no significant difference in sex between dedifferentiated versus non-dedifferentiated chondrosarcoma patients, but patients with dedifferentiated tumors tended to be older than those with non-dedifferentiated tumors (66 vs. 59, *p* = 0.029). Size did not differ significantly between dedifferentiated vs. non-dedifferentiated tumors (12.90 cm vs. 9.32 cm, *p* = 0.065). Tumor location did not demonstrate an obvious predilection for dedifferentiated histology within this cohort; however, interpretation of location-specific trends was limited by small subgroup sizes, particularly among tibial lesions (Table 2).

**Table 2:** Patient demographics and tumor characteristics

Variable	Total	Grade 1	Grade 2	Grade 3	Non-dedifferentiated	Dedifferentiated	*p*-value
All tumors	67	21	18	5	44	23	
Age							0.029*
Mean, yrs	61.36	57.19	61.56	56.50	58.91	66.04	
Range, yrs	18–85	28–75	18–85	24–73	18–85	42–82	
Sex, n (%)							0.39†
Male	33 (49)	5 (24)	11 (61)	4 (80)	20 (45)	13 (57)	
Female	34 (51)	16 (76)	7 (39)	1 (20)	24 (55)	10 (43)	
Tumor location, n (%)							
Femur	44 (66)	12 (57)	12 (67)	4 (80)	28 (64)	15 (65)	
Humerus	19 (28)	8 (38)	5 (28)	1 (20)	14 (32)	5 (22)	
Tibia	4 (6)	1 (5)	0 (0)	0 (0)	1 (2)	3 (13)	
Radius	1 (1)	0 (0)	0 (0)	0 (0)	1 (2)	0 (0)	
Tumor size, cm	10.51	7.47	9.93	14.90	9.32	12.90	0.065*

*Welch’s t-test

†Chi-squared test

Multiple preoperative imaging features showed a significant positive association with dedifferentiated chondrosarcoma on univariate analysis (Table 3). In contrast, of the seven imaging features examined in the final multivariate analysis, only marrow edema was found to independently identify dedifferentiated chondrosarcoma from other intramedullary long bone chondrosarcomas (OR = 8.6, 95% CI 1.7–43, *p* = 0.0091) (Table 4). Marrow edema remained a significant predictor even after controlling for age, sex, and tumor size (*p* = 0.0057). Marrow edema was identified in 20 (87%) of 23 dedifferentiated lesions and only 14 (31.8%) of 44 non-dedifferentiated lesions, suggesting a very high sensitivity in identifying dedifferentiated chondrosarcoma (87%) and a moderately high specificity (68.2%) (Table 3). Positive predictive value of marrow edema equaled 59% and negative predictive value equaled 91%. Cortical thickening was a weak but non-significant positive predictor of dedifferentiated status (OR = 4.7, 95% CI 0.86–26, *p* = 0.074). Additionally, medullary expansion was a weak but non-significant negative predictor (OR = 0.27, 95% CI 0.050–1.5, *p* = 0.13) (Table 4).

**Table 3:** frequency of imaging features in dedifferentiated and non-dedifferentiated chondrosarcoma, n (%)

Imaging feature	All tumors (*n* = 67)	Non-dedifferentiated (*n* = 44)	Dedifferentiated (*n* = 23)	*p*-Value
Pathologic fracture	10 (14.9%)	3 (6.8%)	7 (30.4%)	0.025
> 2/3 Endosteal scalloping	43 (64.2%)	23 (52.3%)	20 (87.0%)	0.0072
Medullary expansion	33 (49.3%)	20 (45.5%)	13 (56.5%)	0.609
Periosteal reaction	29 (43.3%)	14 (31.8%)	15 (65.2%)	0.020
Cortical thickening	23 (34.3%)	12 (27.3%)	11 (47.8%)	0.180
Marrow edema	34 (50.7%)	14 (31.8%)	20 (87.0%)	< 0.001
Soft-tissue involvement	41 (59.7%)	21 (47.7%)	19 (82.6%)	0.0090

*p*-Values are from Fisher’s exact test (2 × 2, univariate analysis)

**Table 4:** Risk factors for dedifferentiated chondrosarcoma on multivariate analysis

Imaging feature	OR	95% CI (Low)	95% CI (High)	Coefficient	*p*-Value
Pathologic fracture	4.911	0.726	33.208	1.592	0.103
> 2/3 Endosteal scalloping	5.277	0.756	36.890	1.635	0.094
Medullary expansion	0.271	0.050	1.481	− 1.315	0.133
Periosteal reaction	3.663	0.783	17.134	1.241	0.099
Cortical thickening	4.690	0.860	25.572	1.526	0.074
Marrow edema	8.586	1.707	43.188	2.263	0.0091*
Soft-tissue involvement	1.444	0.276	7.554	0.324	0.664
Intercept				− 4.398	< 0.001*

* Significant at *p* < 0.05

VIFs for the seven imaging features ranged from 1.28 (pathologic fracture) to 1.84 (medullary expansion) in the multivariate model. Bone marrow edema (VIF = 1.51) showed minimal collinearity with other aggression markers, supporting its independent predictive value. The Hosmer–Lemeshow goodness-of-fit test for the multivariate analysis yielded χ² = 8.5 (degrees of freedom (df) = 8, *p* = 0.39), indicating no evidence of lack of fit.

**Fig. 1:**
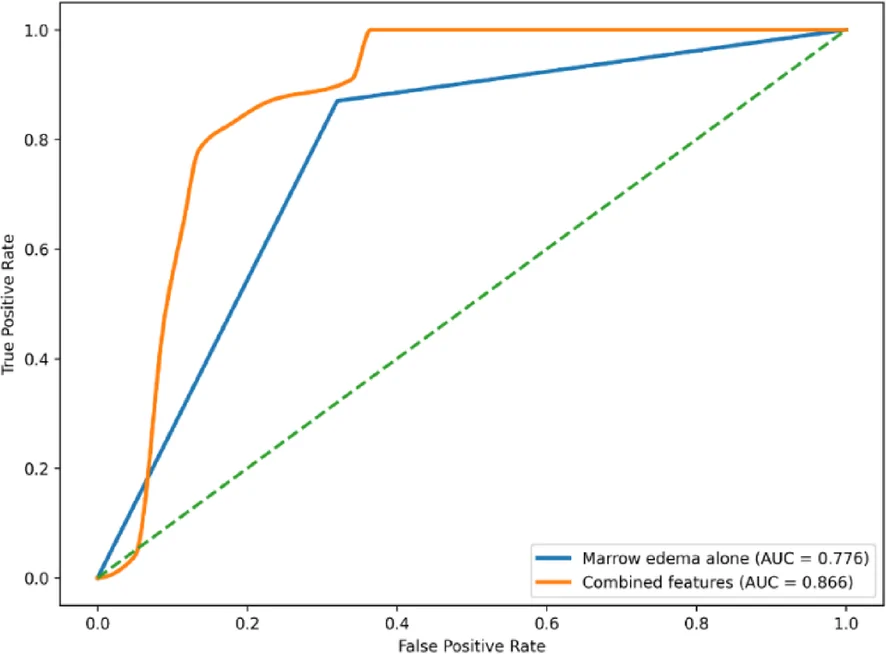
Receiver operating characteristic (ROC) curves demonstrating diagnostic performance of marrow edema alone versus combined imaging features. The area under the curve (AUC) for marrow edema alone was 0.776, while the combined feature model demonstrated improved performance with an AUC of 0.866. The dashed line represents a random classifier (AUC = 0.50)

The discriminative performance of individual imaging features and the overall seven-factor multivariate model were compared using ROC analysis. Marrow edema alone in a univariate regression for prediction of dedifferentiated status had a significantly lower AUC (AUC = 0.78, 95% CI 0.67–0.88) than the overall seven-factor multivariate model (AUC = 0.87, 95% CI 0.83–0.97) (*p* < 0.001) (Fig. 1). Other individual imaging features with the next highest AUCs were soft tissue involvement (AUC = 0.67, 95% CI 0.56–0.79), > 2/3 endosteal scalloping (AUC = 0.67, 95% CI 0.58–0.76), and periosteal reaction (AUC = 0.67, 95% CI 0.55–0.79), which each had significantly lower AUCs than marrow edema (*p* = 0.0027).

## Discussion

This study evaluated nine preoperative radiographic features previously described in the characterization of intramedullary long bone chondrosarcoma [10], with the goal of identifying specific imaging predictors of dedifferentiated chondrosarcoma status. Among the features assessed, marrow edema was the only imaging variable that remained a statistically significant independent predictor after adjustment for other established markers of radiologic aggressiveness, suggesting that it provides unique diagnostic information beyond conventional indicators of tumor grade. This highlights marrow edema as a potentially valuable preoperative biomarker for identifying dedifferentiated chondrosarcoma status among intramedullary long bone chondrosarcoma lesions.

While other imaging features such as cortical thickening demonstrated weak positive associations with dedifferentiation, they did not reach statistical significance in a multivariate model. Additionally, while complete cortical breach was a very sensitive method of detecting dedifferentiated chondrosarcoma, its complete sensitivity in this sample did not allow the multivariate analysis to converge. Finally, the sizeable collinearity between circumferential and cranio-caudal length endosteal scalloping warranted a combination of these two features in the final analysis.

Interestingly, the predictive performance of the full multivariate model (which incorporated all seven imaging features) was slightly better than marrow edema alone, as reflected by a significantly higher AUC. However, marrow edema had a significantly higher AUC compared to any other single imaging variable. It also retained the highest individual sensitivity, appearing in 87% of dedifferentiated cases, while still maintaining a 68.2% specificity. This makes marrow edema a useful initial screening marker, as its absence makes dedifferentiation unlikely, while its presence should prompt further imaging and expedited biopsy. This potential role in biopsy planning is particularly important given the recognized limitations of needle biopsy in cartilaginous tumors. Previous studies have demonstrated substantial discordance between biopsy and final resection histology, with underestimation of tumor grade and failure to identify dedifferentiated components occurring in a meaningful proportion of cases [10, 14, 15]. Saifuddin et al. reported that MRI review identified high-grade or dedifferentiated lesions missed on initial image-guided biopsy with over 90% diagnostic accuracy and specifically recommended repeat biopsy when imaging findings are discordant with biopsy results [14]. Similarly, Vijayakumar et al. found that nearly two-thirds of tumors ultimately found to contain dedifferentiation were not identified as such on preoperative biopsy [15]. Therefore, the presence of marrow edema and other aggressive imaging features should not only raise suspicion for dedifferentiated chondrosarcoma but may also help guide biopsy strategy, including consideration of repeat sampling or targeting of the most radiographically aggressive regions when clinicopathologic findings are discordant. Nonetheless, when feasible, incorporating all seven imaging variables provides the most accurate assessment and may improve diagnostic confidence beyond marrow edema alone.

**Fig. 2:**
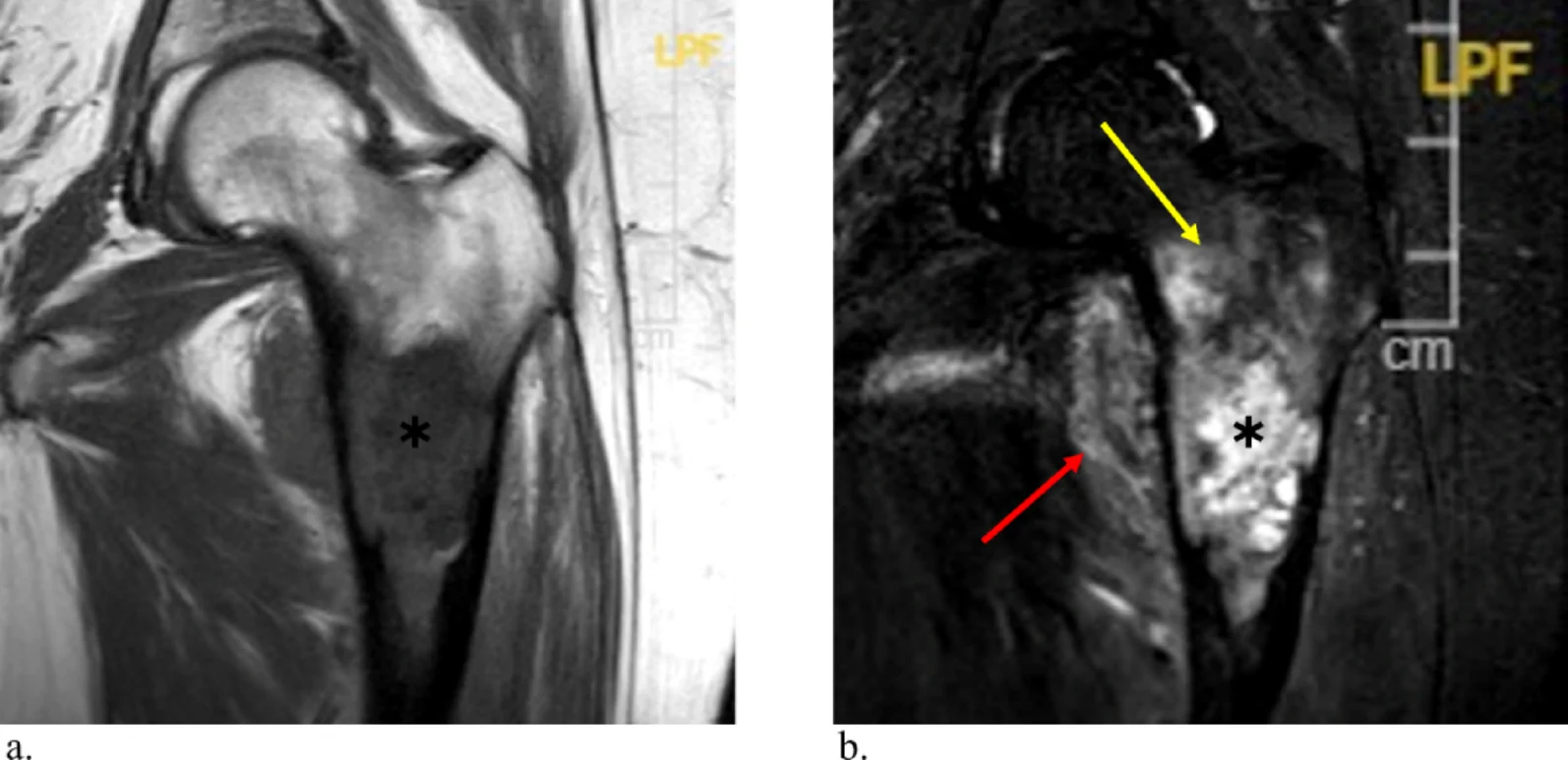
Preoperative imaging in a patient diagnosed with dedifferentiated chondrosarcoma of the left proximal femur confirmed via surgical pathology. Coronal T1-w **a** and coronal STIR **b** MR imaging demonstrates a lobulated markedly STIR hyperintense central cartilaginous neoplasm (*) with surrounding ill-defined T1-w hypointense and moderately STIR hyperintense bone marrow edema (arrow) involving the femoral neck, peritrochanteric and subtrochanteric regions of the proximal femur

**Fig. 3:**
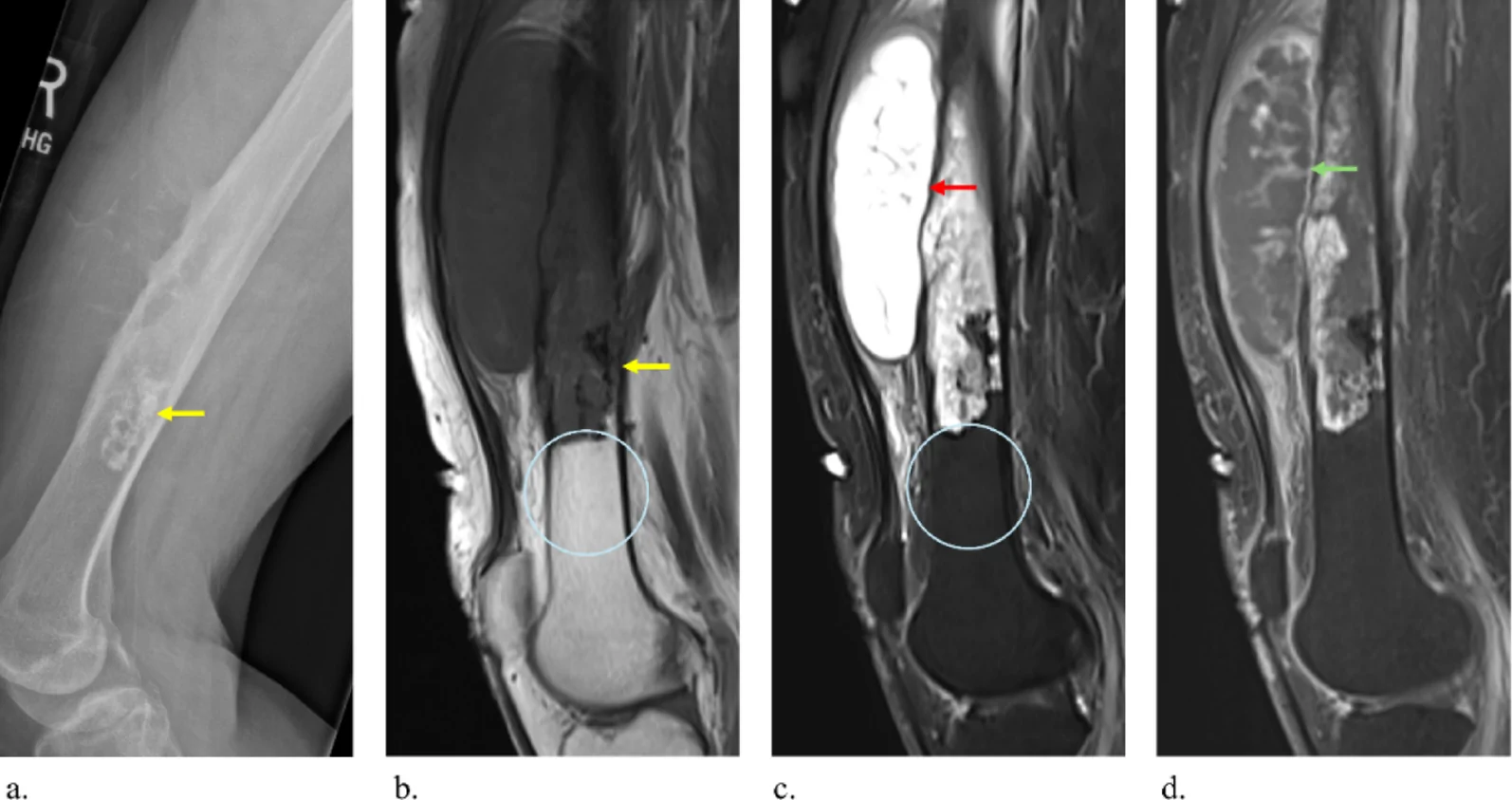
Preoperative imaging in a patient with grade 2 conventional chondrosarcoma of the right femur confirmed via surgical pathology. **a** Lateral radiograph of the right femur demonstrating mildly expansile lucent and mineralized (yellow arrow) medullary bone lesion with mineralized anterior extraosseous soft tissue mass. **b** Sagittal T1-w MR image demonstrating marrow replacing lesion with hypointense mineralization (yellow arrow) and absence of peritumoral bone marrow edema (circle). **c** Sagittal STIR MR image demonstrating markedly hyperintense medullary and extraosseous mass typical of chondroid neoplasm (red arrow) and absence of peritumoral bone marrow edema (circle). **d** Sagittal contrast-enhanced fat suppressed T1-w MR image demonstrating peripheral and internal septal enhancement typical of chondroid neoplasms (green arrow)

Bone marrow edema, often called edema-like marrow signal intensity (ELMSI) in radiology literature [16], should be identified on MRI as an area of high signal intensity on fat-suppressed T2-weighted or short tau inversion recovery (STIR) sequences that extends beyond the margins of the primary tumor defined on T1-weighted imaging, as demonstrated in Fig. 2. This finding should be distinguished from the tumor-reactive zone described around some high-grade sarcomas, which may demonstrate contrast enhancement related to increased vascularity. Conversely, absence of marrow edema shows no intramedullary extension of T2-weighted or STIR MRI signal beyond the tumor border defined on T1-weighted MRI, as demonstrated in Fig. 3 [16–18].

Bone marrow edema can be observed in multiple types of benign and malignant tumors. Benign types include Langerhans cell histiocytosis, osteochondroma, chondroblastoma, and osteoid osteoma, while malignant tumors include chondrosarcoma, Ewing’s sarcoma, and osteosarcoma [19]. While the presence of marrow edema in chondrosarcoma has been previously noted, its specific utility in identifying dedifferentiated chondrosarcoma has not been emphasized in existing literature. Importantly, the present study was restricted to intramedullary long bone lesions. Cartilaginous tumors arising in flat bones such as the pelvis, scapula, and ribs exhibit distinct biological behavior and carry a higher baseline likelihood of high-grade histology compared with appendicular long bone lesions [20–23]. Consequently, the predictive performance of marrow edema observed in this cohort may not be directly generalizable to these anatomical locations, and dedicated investigations in axial and flat-bone chondrosarcomas are warranted.

Studies by Gundavda et al. [10] in 2023 and others have included marrow edema amongst other factors as a radiographic tool to distinguish between low versus high-grade long bone cartilage lesions [24–26]. However, these methods did not isolate independent predictors of dedifferentiated chondrosarcoma, specifically. This distinction is clinically important, as prognosis varies dramatically: patients with grade 2 tumors have a median survival of approximately 100 months, while those with dedifferentiated tumors survive a median of only 11 months [1, 2]. The prognostic implications of early identification are further underscored by the high metastatic potential of dedifferentiated chondrosarcoma. Kozawa et al. in 2022 reported a 29% increase in disease-specific mortality among patients presenting with metastasis compared to those without (94% vs. 65%), emphasizing the importance of timely diagnosis and resection [27].

Additional imaging clues for dedifferentiation have been described, including the presence of bimorphic radiographic patterns. Littrell et al. in 2004 observed that dedifferentiated chondrosarcomas frequently demonstrated adjacent regions of contrasting mineralization, such as a mineralized component bordering a prominent lytic area [12, 13]. From a procedural standpoint, recognition of these biphasic imaging patterns may also aid biopsy planning, as the more aggressive appearing component may represent the dedifferentiated portion of the tumor and therefore constitute the highest-yield target for tissue sampling. While useful, this finding was not validated against non-dedifferentiated lesions, limiting its specificity.

This study has several limitations. Given the small number of grade 3 conventional tumors, this study was not sufficiently powered to assess discrimination between high-grade subtypes. Therefore, larger cohorts are needed to determine whether marrow edema differentiates dedifferentiated from grade 3 chondrosarcoma, specifically. Nonetheless, when compared to the broader population of intramedullary long bone tumors encountered in clinical practice, marrow edema remained an independent predictor of dedifferentiated status. Furthermore, although marrow edema was a statistically significant independent predictor, the wide confidence intervals surrounding several odds ratio estimates likely reflect the modest sample size and warrant validation in larger cohorts. Marrow edema was assessed qualitatively without a predefined thickness threshold. While this approach reflects routine clinical radiologic interpretation, future prospective studies may benefit from standardized quantitative MRI measurements of edema burden to improve reproducibility and determine whether the extent of marrow edema further stratifies the risk of dedifferentiated histology. Contrast-enhanced MRI was not uniformly available throughout the study period, limiting our ability to distinguish edema-like marrow signal from the enhancing tumor-reactive zone described around high-grade sarcomas. Future prospective investigations utilizing standardized contrast-enhanced MRI protocols may better differentiate these entities and clarify their relative contributions to predicting dedifferentiated chondrosarcoma. Additionally, biphasic MRI morphology, a recognized feature of dedifferentiated chondrosarcoma, was not systematically evaluated because of heterogeneous imaging protocols and inconsistent availability of imaging sequences across the study period. Future studies should investigate the combined diagnostic utility of marrow edema, contrast-enhancement patterns, and biphasic imaging characteristics. The exclusion of extraosseous and periosteal chondrosarcomas restricts the applicability of these findings to intramedullary lesions. Because the cohort was restricted to surgically resected intramedullary long bone tumors at a tertiary referral center, selection bias is possible, with relative enrichment of aggressive lesions. Finally, because the analysis was limited to intramedullary long bone tumors, the results may not generalize to lesions of the pelvis, scapula, ribs, or axial skeleton.

## Conclusions

Bone marrow edema on MRI is a highly sensitive, independently predictive feature of dedifferentiated chondrosarcoma of the long bones, offering a valuable biomarker to enhance early screening. Although not diagnostic in isolation and best interpreted alongside additional imaging variables, its presence in 87% of dedifferentiated cases compared with 32% of non-dedifferentiated tumors underscores its clinical utility in raising concern for this aggressive malignancy, which carries a median survival of approximately 11 months. Recognition of marrow edema may therefore prompt expedited biopsy and surgical intervention, with the potential to improve outcomes in this rapidly progressive disease. Prospective, multi-center validation is warranted, and future studies should explore marrow edema’s applicability to chondrosarcomas in other anatomical sites, including the pelvis and axial skeleton.

## Data Availability

The datasets used and/or analyzed during the current study are available from the corresponding author on reasonable request.

## Abbreviations

AUC: Area under the curve
CI: Confidence interval
CT: Computed tomography
df: Degrees of freedom
ELMSI: Edema-like marrow signal intensity
MRI: Magnetic resonance imaging
OR: Odds ratio
ROC: Receiver operating characteristic
SEM: Standard error of the mean
STIR: Short tau inversion recovery
VIF: Variance inflation factor
